# Features of diclofenac biodegradation by *Rhodococcus ruber* IEGM 346

**DOI:** 10.1038/s41598-019-45732-9

**Published:** 2019-06-24

**Authors:** Irina B. Ivshina, Elena A. Tyumina, Maria V. Kuzmina, Elena V. Vikhareva

**Affiliations:** 1 Institute of Ecology and Genetics of Microorganisms, Ural Branch of the Russian Academy of Sciences, 13 Golev Street, 614081 Perm, Russia; 2 Perm State National Research University, 15 Bukirev Street, 614990 Perm, Russia; 3 Perm State Pharmaceutical Academy, 2 Polevaya Street, 614990 Perm, Russia

**Keywords:** Applied microbiology, Bacteria, Environmental microbiology

## Abstract

This study investigated the ability of rhodococci to biodegrade diclofenac (DCF), one of the polycyclic non-steroidal anti-inflammatory drugs (NSAIDs) most frequently detected in the environment. *Rhodococcus ruber* strain IEGM 346 capable of complete DCF biodegradation (50 µg/L) over 6 days was selected. It is distinguished by the ability to degrade DCF at high (50 mg/L) concentrations unlike other known biodegraders. The DCF decomposition process was accelerated by adding glucose and due to short-term cell adaptation to 5 µg/L DCF. The most typical responses to DCF exposure observed were the changed ζ-potential of bacterial cells; increased cell hydrophobicity and total cell lipid content; multi-cellular conglomerates formed; and the changed surface-to-volume ratio. The obtained findings are considered as mechanisms of rhodococcal adaptation and hence their increased resistance to toxic effects of this pharmaceutical pollutant. The proposed pathways of bacterial DCF metabolisation were described. The data confirming the C-N bond cleavage and aromatic ring opening in the DCF structure were obtained.

## Introduction

In recent years, there is a growing interest to study the bioavilability and toxic effects of pharmaceutical pollutants on environmental microorganisms, which act as primary response systems and trigger the adaptive reactions. The present study relies on the assumption that members of the genus *Rhodococcus* could play important ecological roles in biological detoxification and decontamination of soils and water. They are stable inhabitants of polluted soils, water bodies, activated sludge, and wastewater; they have high oxidoreductase activities, abundant adaptation abilities for various toxic compounds, and high bioremediation potential for contaminated environments^1–4^. The number of studies on the topical issue is increasing, that indicates the timeliness of exploiting the metabolic *Rhodococcus* capabilities to biodegrade pharmaceuticals^5–10^. We have previously demonstrated for rhodococci to completely biodegrade analgesic and antispasmodic drugs, including paracetamol^11^, drotaverine hydrochloride^12,13^. In the present study, an “exploratory” analysis of their possible role as key biooxidizers of pharmaceuticals from the group of non-steroidal anti-inflammatory drugs (NSAIDs) ubiquitously detected in the environment was in focus^14,15^.

One of such anthropogenic micropollutants is diclofenac (DCF), a phenylacetic acid derived NSAID widely available and used in the world human medicine and veterinary. The annual DCF consumption is expressed in hundreds of tons^16^. Actual concentrations of DCF frequently detected in ground water^17,18^, surface (including marine)^19–22^, sewage^23–27^ and even drinking^28,29^ waters range from 0.02 ng/L to 20.00 μg/L worldwide. Along with non-metabolized DCF, its metabolites (4′-hydroxy-DCF, 5-hydroxy-DCF and *p*-benzoquinone imine of 5-hydroxy-DCF) have been detected in sewage water and river sediments^30,31^.

Because the *n*-octanol/water partition coefficient (log K_ow_) for DCF is 4.51^32^, the lipophilicity of this chemical can contribute to its potential bioaccumulation in living organisms, and first of all in hydrobionts. The Hazard Quotient values for DCF detected in freshwater far exceed 1 (HQ 155.22)^33^. It indicates that this pharmaceutical substance is a source of adverse effects on natural environments (hydrobionts and humans)^34^. DCF is on the Watch List of substances of particular concern in the EU, and has been considered as a pharmaceutical of the greatest potential environmental risk^35^.

Application of traditional physicochemical technologies for removal of pharmaceutical pollutants from water/wastewater is eco-unsafe because it is limited by possible by-products of their degradation, and is fairly expensive due to high operational and energy costs^36–38^. In terms of efficiency, safety and economic criteria, the biotechnological ways of converting these environmental stressors are recognized as priority approaches.

However, studies on DCF bioconversion are still few and mainly focused on eukaryotic organisms, in particular basidiomycetes (*Bjerkandera*, *Trametes*, *Phanerohaete*), zigomycetes (*Cunninghamella*) and entomopathogenic (*Beauveria*) fungi^39^. The bacterial degradation of DCF is little understood, except for the previous occasional reports on DCF biotransformations by Gram-positive bacteria *Actinoplanes*^40^ and *Brevibacterium*^41^, as well as by microorganisms of activated sludge^42–44^. More recently, Moreira *et al*.^45^ reported for the first time the complete DCF degradation using an alphaproteobacterial strain *Labrys portucalensis* F11 in the presence of acetate and proposed possible metabolic pathways for DCF decomposition. Biodegradation of DCF at relatively high concentrations have been demonstrated using enterobacterial *Klebsiella* sp. and *Enterobacter hormaechei*^46,47^. Meanwhile, there is a clear lack of information on the mechanism and effectiveness of protective responses triggered in the bacterial cell exposed to this ecotoxicant.

The purpose of this study was to assess the resistance of actinobacteria of the genus *Rhodococcus* to DCF exposure and their capabilities for its bioconversion, to investigate the features of bacterial cell-pharmaceutical interactions. This study has investigated the patterns of DCF biodegradation in relation to physiological states and culture conditions of rhodococci, as well as their responses to adverse ecotoxicant effects. The metabolites formed in the degradation process have been detected and reactions of bacterial DCF degradation have been described. This is the first report on the ability of *Rhodococcus* representatives to degrade this ecotoxic compound.

## Materials and Methods

### Microorganisms

In this work, 104 rhodococcal strains from the Regional Specialised Collection of Alkanotrophic Microorganisms (IEGM, WDCM 768, http://www.iegmcol.ru) were used. They belonged to 10 *Rhodococcus* species: *R. cercidiphylli* (1 strain), *R. corynebacterioides* (2 strains), *R. erythropolis* (41 strains), *R. jostii* (3 strains), *R. koreensis* (1 strain), *R. pyridinivorans* (2 strains), *R. qingshengii* (4 strains), *R. rhodochrous* (8 strains), *R. ruber* (41 strains), and *R. wratislaviensis* (1 strain). The strains were selected by geography and isolation sources as well as by the known catalytic activities of rhodococci towards complex organic compounds^48^.

### Chemicals

Diclofenac (C_14_H_11_Cl_2_NO_2_, CAS number 15307-86-5, 2-[2-(2,6-dichloroanilino)phenyl]acetic acid as the sodium salt) was used in the form of a pharmaceutical substance (white or light-beige crystalline odorless powder, 99.0% purity in terms of dry matter, sparingly soluble in water; Kairav Chemicals Ltd, India). Chemicals, including ibuprofen, meloxicam, acetonitrile and chloroform, were of chemical, analytical or extra-pure grades (Kryochrom, Russia; Merck, Germany; Sigma-Aldrich, USA). A Millipore Simplicity Personal Ultrapure Water System (Millipore, USA) was employed to obtain ultrapure water for high-performance liquid chromatography (HPLC).

### Culture conditions

Mineral medium RS^48^ was used in DCF biodegradation experiments. The initial DCF concentrations (50 μg/L and 50 mg/L) were chosen with regard for the large amounts to be used, its release rate into the environment, and ecological persistence. The selection relied on the actual DCF concentrations detected in aquatic and soil environments, and on the assumption that large doses cause negative effects, but also stimulate the organism’s defense mechanisms, while small doses cause negative effects without stimulating the defenses. DCF was used as 0.1% water solution sterilized at 110 °C for 15 min. Before inoculation, the initial pH of the medium was 7.0. Cells pre-grown in LB broth (Sigma-Aldrich, USA) for two days and washed twice with phosphate buffer (pH 7.0) were inoculated into the RS medium to the final concentration of 3.7 × 10^7^ cells/mL. Rhodococci were pre-grown at a low (5 μg/L) DCF concentration. D-Glucose (0.5%) was used as a co-substrate. Biodegradation was carried out at 28 °C. To avoid DCF photooxidation, flasks were protected by light-proof material. Due to lengthy (more than 60 days) laboratory experiments, the obtained values were corrected for water evaporation using equations in Gauthier *et al*.^5^.

### Controls

The controls were (i) mineral medium plus DCF (to assess the abiotic degradation of DCF); (ii) mineral medium with DCF plus rhodococci inactivated by threefold autoclaving at 120 °C for 20 min (to evaluate DCF adsorption on bacterial cells); (iii) mineral medium plus glucose and living bacterial cells without DCF (to discriminate DCF decomposition metabolites).

### Determination of the minimal inhibitory concentrations of DCF

The minimal inhibitory concentrations (MICs) of DCF for rhodococci were determined by a twofold dilution method^49^ in LB broth using standard 8 × 12 (96) polystyrene plates. DCF was added at the initial concentration of 200 mg/L to the first well in each row, followed by a twofold dilution. The resulting mixture was supplemented with 10 μL of the bacterial test culture (3 × 10^7^ cells/mL) pre-grown in LB broth for 2 days. The bacteria were incubated at 28 °C for 3 days and stirred (300 rpm) on a Titramax 1000 plate shaker (Heidolph, Germany), followed by cell staining with 0.2% aqueous iodonitrotetrazolium chloride solution. To determine MICs for mixtures of DCF and other pharmaceuticals (particularly ibuprofen, meloxicam) most frequently detected in the environment, the test compounds were mixed in aliquots.

### Microscopic examinations

Cells were visualized using an Axiostar plus optical microscope (Carl Zeiss, Germany) in a phase contrast mode. Images were captured with the Pixera PRO 150ES camera (Pixera, USA) and analyzed using VideoTesT-Size 5.0 (Akond, Russia). DCF effects on cell morphology and surface relief were examined by a combined atomic-force and confocal laser scanning system consisting of a MFP-3D-BIO™ atomic force microscope (AFM, Asylum Research Inc., USA), and an Olympus Fluo View 1000 confocal laser microscope (CLSM, Olympus Corporation, Japan). To differentiate living and dead cells, the bacterial suspension was stained with LIVE/DEAD^®^ *Bac*Light™ Bacterial Viability Kit (Invitrogen, USA). The root-mean-square roughness of the cell surface, cell length and width were determined, and the cell volume and surface area were calculated. The images were processed using the Igor Pro 6.22 A (WaveMetrics, USA).

### Cell hydrophobicity

The degree of bacterial cell hydrophobicity was determined by the salt aggregation test method^50^. Rhodococci were suspended in a phosphate buffer supplemented with ammonium sulfate. The minimal concentration of (NH_4_)_2_SO_4_ solution, at which cell aggregates were formed, was taken as the conditional value of cell wall hydrophobicity. The degree of hydrophobicity was assessed according to the scale: high hydrophobicity corresponded to the salinity index of the ammonium sulfate solution ranging from 0 to 0.8 M; moderate – from 1.0 to 2.0 M; weak – from 2.2 to 3.8 M^51^.

### Zeta potential measurements

Zeta potential of bacterial cells was measured by dynamic light scattering method using a ZetaSizer Nano ZS analyzer (Malvern Instruments, UK) with the Malvern ZetaSizer software, v. 2.2. Cells grown in the mineral medium plus DCF were washed twice and resuspended in 0.1 M KNO_3_ (pH 7.0) until OD_600_ was 0.2. Measurements were carried out in a U-shaped cuvette with gold-plated electrodes at 25 °C and pH 7.0.

### Total lipid content

Total lipids were determined in comparative studies of 3-day LB-grown bacterial cells and 15-day cells grown in the mineral medium plus DCF. For this, 50 mg of dry cell biomass was aged for 24 h in 5 mL of the following mixture: distilled water (1 mL), chloroform + methanol (4 mL) in a volume ratio of 1:2. After 24 h, the mixture was centrifuged at 3,000 rpm for 10 min. The supernatant was decanted into a centrifuge tube. The resulting precipitate was re-extracted with 5 mL of chloroform-methanol-water (1:2:0.8). The supernatant and the extract supplemented with 5 mL of chloroform-water (1:1) were combined and centrifuged. Aliquots of the chloroform layer were transferred into dry, pre-weighed round-bottomed flasks and diluted with an equivalent amount of benzene. The resulting mixture was evaporated on a rotary evaporator (Heidolph, Germany) at 60 °C to a constant weight. The total lipids were measured gravimetrically and the amount was expressed as percent of absolute dry weight (ADW).

### Respiration measurements

Cell respiratory activity was measured with a 6-channel Micro-Oxymax^®^ respirometer (Columbus Instruments, USA). The experiments were carried out in 300 mL Micro-Oxymax glass bottles with 100 mL of the mineral medium at constant stirring (300 rpm, 28 ± 2 °C). The amounts (μL) and rates (μL/min) of O_2_ consumed and CO_2_ released were measured. The respiratory activity was automatically recorded every 42 min for 5‒10 days.

### Analytical methods

DCF concentration was HPLC-measured using a LC Prominence chromatograph (Shimadzu, Japan) equipped with a Phenomenex Jupiter^®^ 5 u reversed-phase C18 300, A column 250 × 4.6 mm, 5 micron (Phenomenex, USA), and a diode-matrix detector. Mobile phase: phosphate buffer (pH 3.5):acetonitrile (60:40, v/v). Elution mode was isocratic. Mobile phase flow rate was 1.0 mL/min. Detection wavelength was at 273 nm. Volume of the injected sample was 20 μL. Thermostat temperature was 40 °C. The DCF retention time was 18.7 ± 0.02 min. Samples for the analysis were centrifuged at 10,000 rpm for 5 min. The supernatant was filtered through a 0.20 μm pore size membrane filter (Whatman, UK).

DCF degradation products were analyzed by gas chromatography-mass spectrometry (GC-MS) using an Agilent 6890–5973 N chromatograph (Agilent Technologies, USA) equipped with a HP-5MS capillary column (30 m; inner diameter, 0.25 mm) operating in the ionization mode with an electronic impact at 70 eV. Helium was the carrier gas (1 mL/min). Injector and interface temperatures were 260 and 290 °C, respectively. Column temperature: 130 °C/10 °C/min/300 °C. The chloroform extract (1 μL) was injected without separation of the carrier gas flow. The mass spectra were recorded in the range of 40–500 *m/z*. The resulting mass spectra were compared with NIST 98 Mass Spectral Library spectra with the Microsoft^®^ Windows Search Program (Version 1.7). Mass spectra of the test substance were considered as identified when they were identical to library spectra by more than 90%. To isolate DCF and its possible metabolites, the culture medium was acidified with 10% aqueous HCl solution to pH 2.0 and extracted three times with an equal volume (10 mL) of chloroform. The combined extracts were dried over Na_2_SO_4_. The solvent was removed on a Laborota 4000 efficient rotary evaporator (Heidolph, Germany). Phenylacetic and fumaric acids were identified by thin layer chromatography (TLC) in the system of benzene:95% ethanol:glacial acetic acid (45:8:4, v/v/v) using Sorbfil plates (Russia). The infrared (IR) spectra of dry residues obtained after evaporation of the mixture of DCF biodegradation products were measured in KBr pellets on a SPECORD M-80 IR spectrophotometer (Carl Zeiss Jena, Germany).

## Results

### Rhodococci resistance to DCF

**Figure 1:**
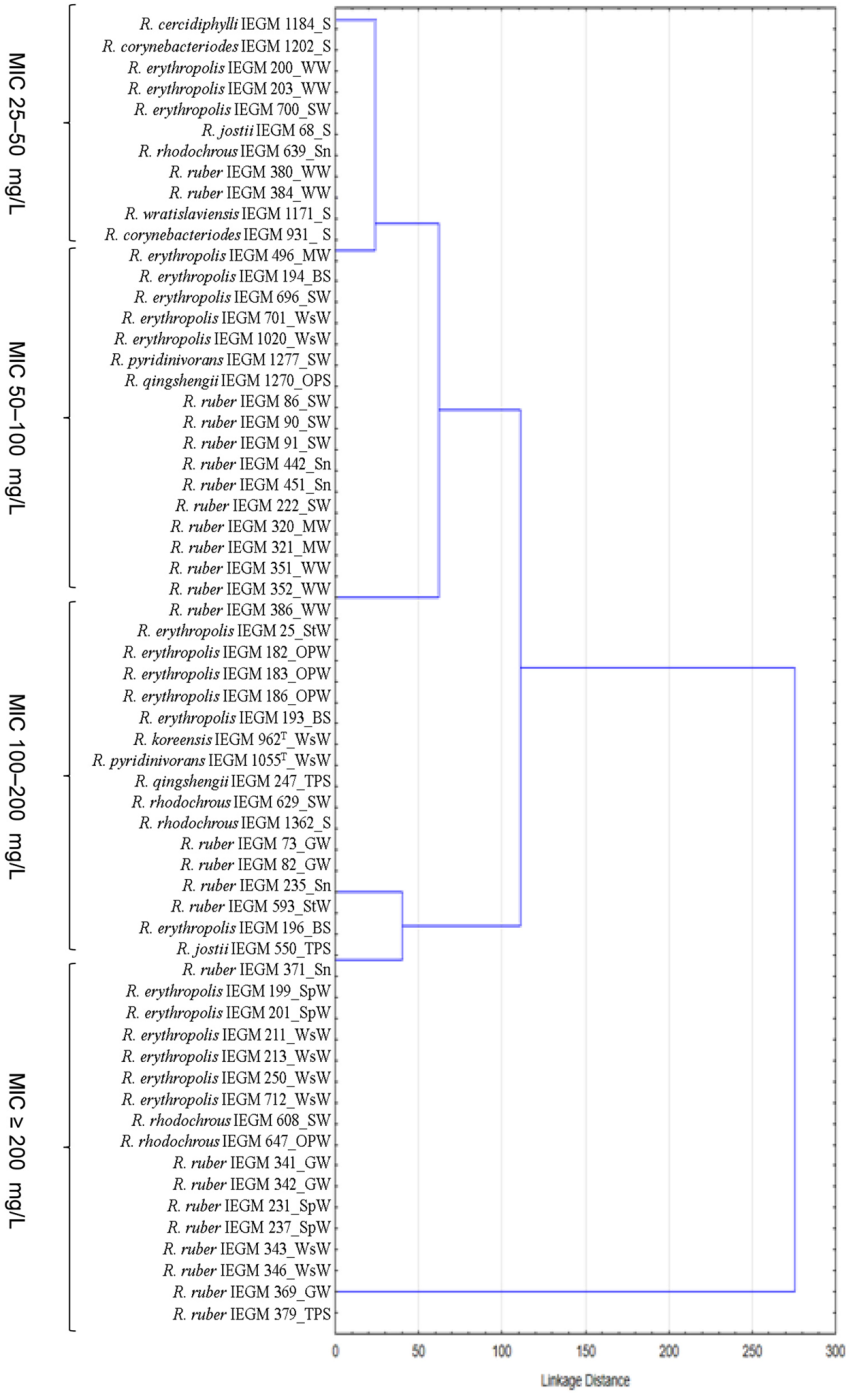
Dendrogram illustrating the resistance to DCF of rhodococci isolated from various ecological systems. BS – bottom sediments, GW – ground water, MW – mineral water, OPS – oil-polluted soil, OPW – oil-polluted water, S – soil, Sn – snow, SW – surface water, SpW – spring water, StW – stratal water, TPS – technogenecally polluted soil, WW – well water, WsW – waste water. All MIC results were ±1 log_2_ dilution step for DCF.

The great majority of the cultures tested retained their viability when exposed to DCF concentrations ranging from 50 to 80 mg/L. The dendrogram (Fig. 1) presents the clustering of bacterial strains according to DCF MICs. Rather few (18) strains resisted relatively high (MIC ≥200 mg/L) DCF concentrations. The most resistant appeared to be strains from ecologically important species *R*. *erythropolis*, *R*. *rhodochrous* and *R*. *ruber*. They were previously isolated mainly from municipal wastewater (*R*. *erythropolis* IEGM 211, IEGM 213, IEGM 250, IEGM 712; *R*. *ruber* IEGM 343, IEGM 346), spring water (*R*. *erythropolis* IEGM 199, IEGM 201; *R*. *ruber* IEGM 231, IEGM 237) and groundwater (*R*. *ruber* IEGM 341, IEGM 342, IEGM 369).

According to the published data, DCF MIC values for pathogenic and opportunistic strains of *Escherichia coli* C600 and *Staphylococcus aureus* 6571 were 50‒100 mg/L^52^, *Enterococcus faecalis* −50 mg/L^53^, *Mycobacterium* spp. −10‒25 mg/L^54^. Our results indicate that environmentally isolated *Rhodococcus* strains resisted higher (MIC ≥200 mg/L) DCF concentrations.

The least (MIC 25‒50 mg/L) DCF resistant strains were isolated from surface water (*R. erythropolis* IEGM 700; *R*. *pyridinivorans* IEGM 1277; *R*. *ruber* IEGM 86, IEGM 90, IEGM 91, IEGM 222), well water (*R*. *erythropolis* IEGM 200, IEGM 203; *R*. *ruber* IEGM 351, IEGM 352, IEGM 380, IEGM 384), mineral water (*R. erythropolis* IEGM 496; *R*. *ruber* IEGM 320, IEGM 321), soil (*R*. *cercidiphylli* IEGM 1184; *R*. *corynebacterioides* IEGM 931, IEGM 1202; *R*. *jostii* IEGM 68; *R*. *wratislaviensis* IEGM 1171), and snow (*R*. *rhodochrous* IEGM 639; *R*. *ruber* IEGM 442, IEGM 451).

In aquatic ecosystems, a mixture of pharmaceuticals from different therapeutic groups, including NSAIDs, is usually found^17,19,21,23^. Analysis of DCF mixed with ibuprofen and meloxicam showed that the cocktail of these substances (even at lower concentrations) had a greater inhibitory effect on *R*. *ruber* IEGM 346 cells than each compound separately. According to our data, DCF MIC value was 200 mg/L, while it decreased eightfold (to 25 mg/L) in a mixture with ibuprofen and meloxicam (Table 1). The obtained findings are consistent with other studies reporting that a pharmaceutical cocktail at lower concentrations causes greater ecotoxicological damage than individual compounds^32^.

**Table 1:** Minimal inhibitory concentrations (MICs) of individual NSAIDs and their mixtures.

Strain	MIC, mg/L					
	DCF	IBU	MLX	DCF + IBU + MLX	DCF + IBU	DCF + MLX
*R. ruber* IEGM 346	≥200	500	≥300	25	50	100

DCF – diclofenac, IBU – ibuprofen, MLX – meloxicam.

To study DCF as a metabolic factor in rhodococci, *R*. *ruber* IEGM 346 (GenBank KF155234) was selected. It is characterized by pronounced emulsifying and biodegrading abilities for individual hydrocarbons and petroleum products, and by its resistance to Cu^2+^, Mo^6+^ and Pb^2+^ (5.0 mM). Additionally, this strain exhibits stable activity in extreme acidity (pH 2‒6) and salinity (2‒6% NaCl) of the medium^48^.

### DCF biodegradation by R.*ruber* IEGM 346

**Figure 2:**
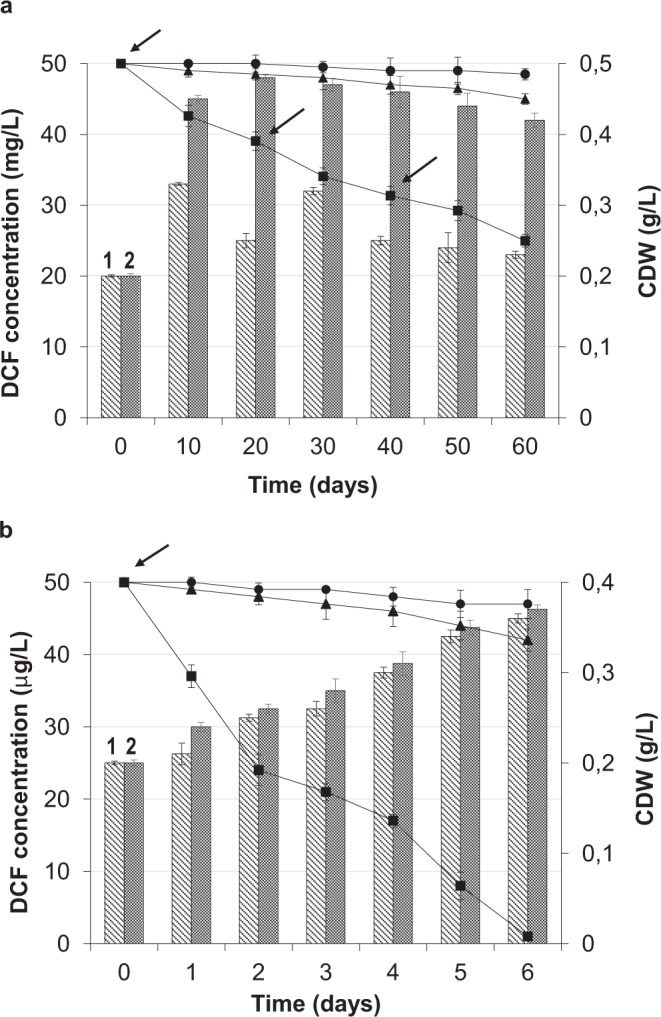
Biodegradation of 50 mg/L (**a**) and 50 μg/L (**b**) DCF by *R. ruber* IEGM 346 (■) in the presence of glucose. (●) abiotic control, (▲) biosorption control, (1) cellular dry weight of rhodococci in the presence of DCF and glucose, (2) cellular dry weight of rhodococci in the presence of glucose. Arrows indicate the addition of glucose. The error bars represent standard deviations (n = 3).

DCF decomposition in a high (50 mg/L) concentration progressed slowly, indicating the high chemical stability of the parent ecotoxicant molecule (Fig. 2a). The residual DCF in the post-fermentation medium of strain IEGM 346 was yet about 50% on day 60. The average rate of DCF biodegradation was 0.4 mg per day throughout the experiment. The maximum (0.7 mg per day) biodegradation rate was observed during the first 10 experimental days. The maximum specific rate of DCF degradation (mg DCF/mg CDW per day) was 0.02 day^−1^. On day 10, an increased rhodococcal growth and a gradual decrease in DCF concentration were recorded under condition of fractional feeding with glucose as an easily utilized carbon source. Measurable abiotic degradation of DCF ranged from 2 to 5%. The control with inactivated cells (Fig. 2) showed an insignificant (up to 10%) decrease in the initial DCF concentration, indicating a possible partial sorption of the compound on the bacterial cell surface.

For low (50 μg/L) DCF concentration, the situation was different. In that case, a significant DCF loss was recorded within the first two days (Fig. 2b). The average biodegradation rate was 8.3 μg per day. Against the background of achieving the maximum (13 μg/day) rate of DCF biodegradation, a gradual stable increase in cellular biomass occurred. On day 5, an increase in rhodococcal numbers was accompanied by a significant DCF loss, with its complete decomposition being achieved on day 6 of the experiment.

**Figure 3:**
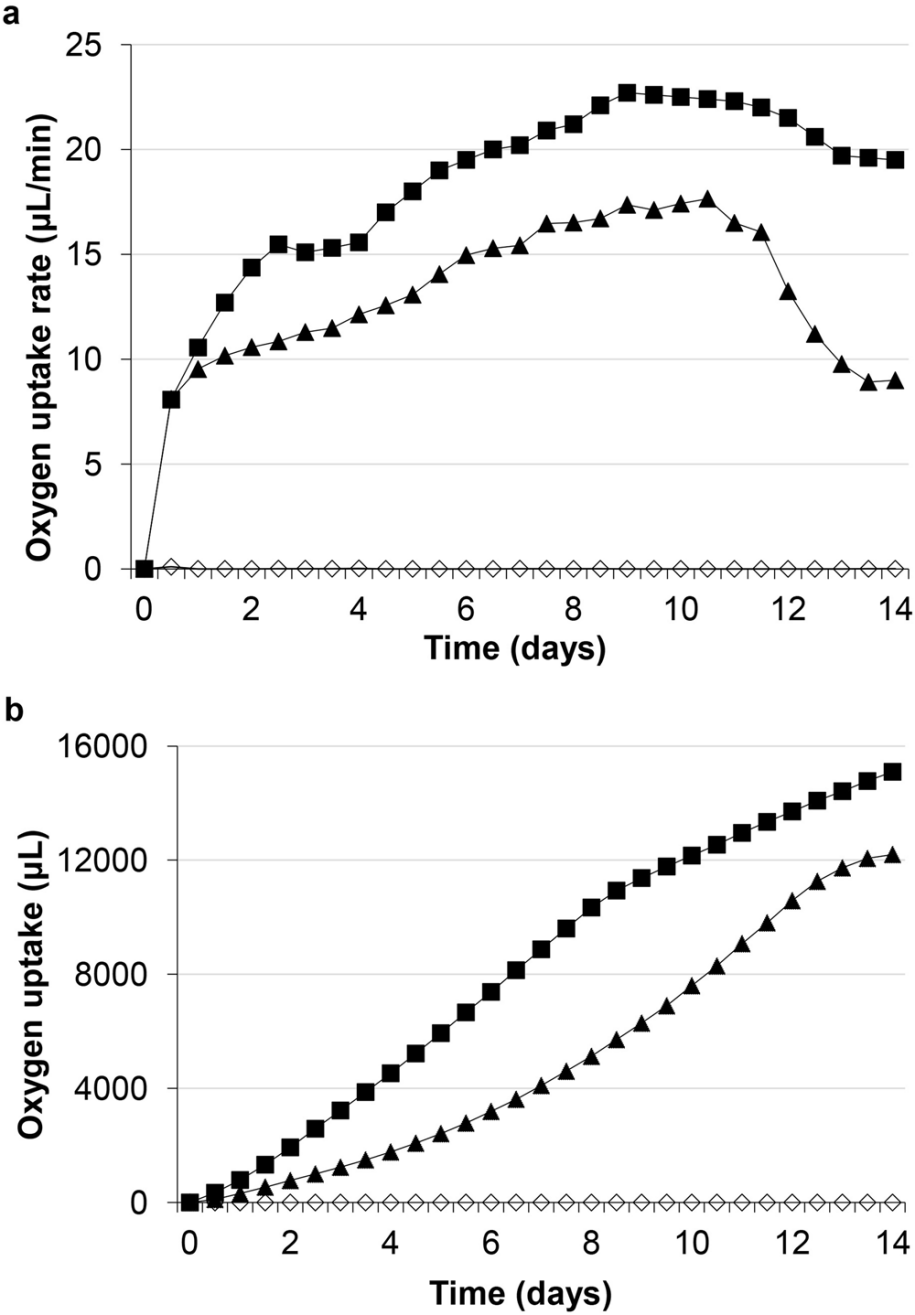
Respiratory activity of *R. ruber* IEGM 346 cells during DCF degradation (■). (**a**) Oxygen uptake rate, (**b**) total oxygen uptake. (▲) biotic control, (◊) abiotic control.

The obtained data were supported by *Rhodococcus* respiratory activity measurements, indicative of cell viability and metabolic process intensity (Fig. 3). Cell respiration in the presence of DCF was significantly higher than in controls. The calculated average (18.9 μL/min) rates of O_2_ consumption during DCF bioconversion were 1.5 times higher than those (12.6 μL/min) in the control (Fig. 3a). The maximum (22.7 μL/min) values of O_2_ consumption rate in the presence of DCF were registered on experimental day 10. After 10 days, the control exhibited a significant decrease in the O_2_ uptake rate, while with DCF there was only a slight decline. Cells consumed 15,930 μl of O_2_ after 14 experimental days, whereas they consumed 12,095 μl of O_2_ with glucose alone (Fig. 3b). In the abiotic control, respiration values were zero. These data confirm that the DCF decomposition was completely dependent on the cell catalytic activity and catalyzed by oxygenases.

### Morphometric changes in rhodococci exposed to different DCF concentrations

**Figure 4:**
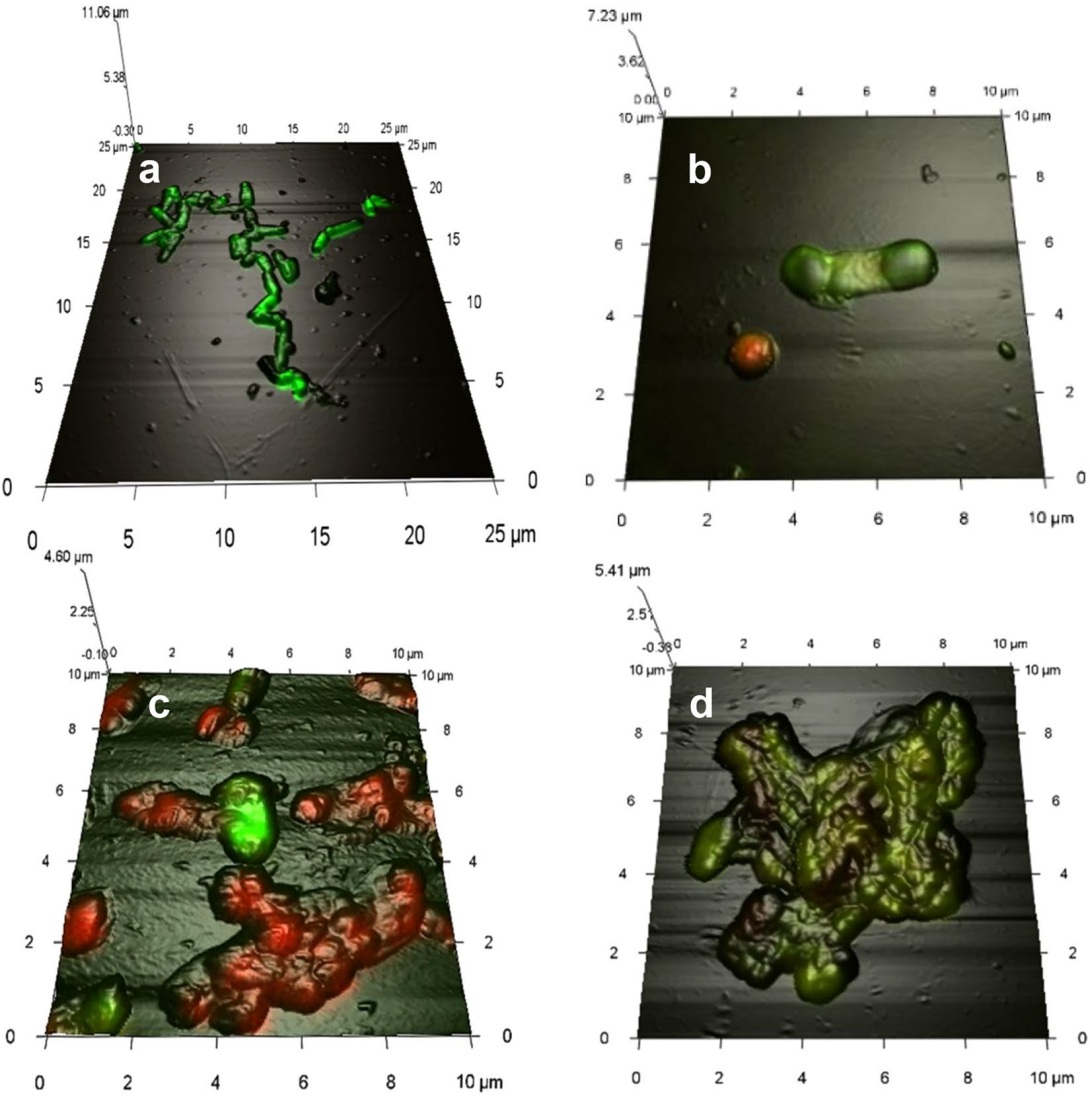
Combined 3D AFM/CLSM images of *R. ruber* IEGM 346. (**a**) Cells grown in LB broth for 2 days; (**b,d**) cells grown in the presence of 50 μg/L DCF for 10 days; (**c**) cells grown in the presence of 50 mg/L DCF for 10 days. Red fluorescence indicates damaged cells. Here and in Figs 5, 6 cells were at the beginning of the stationary growth phase.

**Figure 5:**
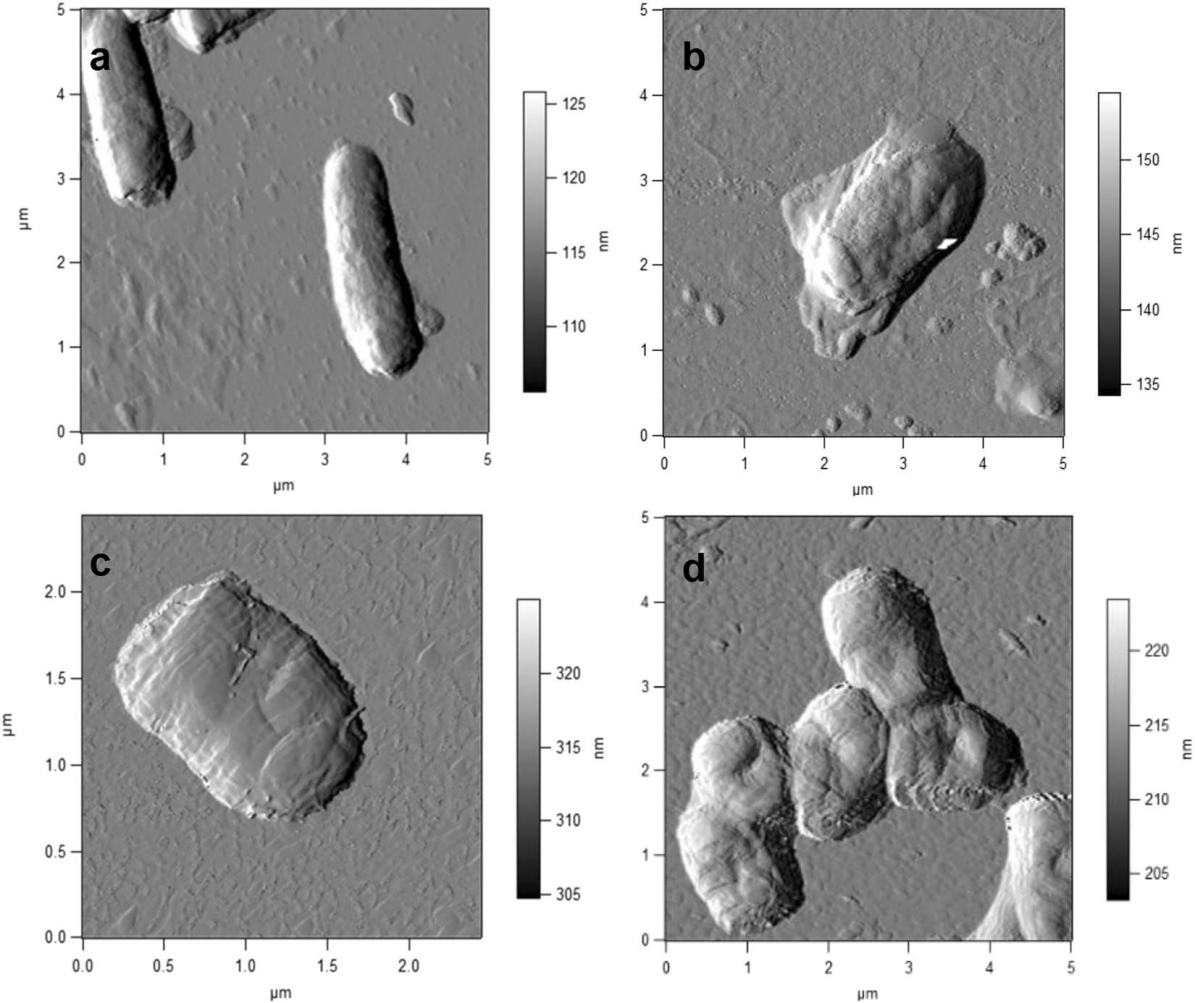
AFM images of *R. ruber* IEGM 346. (**a**) cells grown in LB broth for 2 days; (**b,c**) cells grown in the presence of 50 mg/L DCF for 10 days; (**d**) cells grown in the presence of 50 μg/L DCF for 10 days.

The most typical response of rhodococcal cells exposed to DCF was the formation of separate, different-sized, odd-shaped, multi-cellular aggregates in the liquid medium (Fig. 4c,d). The “cooperative cell system” seems to ensure the consistent functioning of numerous aggregated cells, allows the population to adapt and grow under “harsh” conditions, in which single cells are not able to replicate and biodegrade this ecotoxicant. The maximum (and more extensive) aggregation was observed in the presence of high (50 mg/L) DCF concentration on day 15 of the experiment (Fig. 4c). Under those conditions, the maximum level of distortion in the morphological cell structure was observed: the changed shape (pleomorphic rods) and enlarged average size of vegetative cells mainly due to swelling (Fig. 5c) and the length change, as well as the increased cell surface area compared to the controls (Table 2). Single cells often suffered the damaged integrity of the peptidoglycan layer accompanied by the release of cytoplasmic components into the medium and accumulation of dead cells in the sample (Fig. 5b). At the morphological level, the destructive DCF effect on *R*. *ruber* IEGM 346 was manifested as a decrease (p < 0.001) in the surface-to-volume ratio (S/V) of bacterial cells. The reduction in S/V appears to play an important role in bacterial “opposition” to toxic effects of the contaminant through reducing the cell surface open to contact with the eco-stressor^55^. The obtained results support indirectly the toxic DCF effects on rhodococci. Examination of the surface micro-geometry of living IEGM 346 cells did not reveal significant changes either in the root-mean-square roughness values (variations in values did not exceed 9%) or in the amplitude of the microrelief (Table 2), indicating the serious changes in the cell exposed to high DCF concentrations. In the presence of a higher DCF concentration, the cells were severely damaged (Fig. 4c), whereas at a lower concentration, they retained high viability (Fig. 4d).

**Table 2:** Comparative morphometric characteristics of *R*. *ruber* IEGM 346 incubated in LB broth (control) and DCF-added mineral medium.

Treatment	Length, μm	Width, μm	Volume (V), μm^3^	Surface area (S), μm^2^	Surface area/volume (S/V), μm^−1^	Root-mean-square roughness, nm
Control^a^	3.0 ± 0.02	0.9 ± 0.05	1.9 ± 0.03	5.5 ± 0.05	2.9 ± 0.02	197.8 ± 2.30
50 μg/L DCF^b^	3.5 ± 0.13	1.1 ± 0.02	3.3 ± 0.05	7.9 ± 0.10	2.4 ± 0.08	216.1 ± 5.51
50 mg/L DCF^b^	2.2 ± 0.05	0.8 ± 0.01	1.0 ± 0.02	3.6 ± 0.03	3.6 ± 0,02	249.6 ± 6.64

Cells were cultivated for^a^ 48 h and^b^ 240 h.

At lower (50 µg/L) DCF concentrations, the S/V values of bacterial cells evidenced the opposite: under carbon deficiency, the contact area of rhodococci and DCF increased to enhance its absorption and consumption. In that case, a significant increase in the degree of the cell surface roughness was observed (value variations exceeded 60%) (Table 2). We earlier found a similar response of rhodococci exposed to other xenobiotics: organic contaminants^56^ and resin acids^57^. At lower DCF concentrations, the number of morphologically heterogeneous cells increased: they were smaller in size, some very significantly; became more oval, often heavily swollen or bean-like, with convex edges (Fig. 4b). Most cells revealed structural crater-like relief (Fig. 5d).

In the presence of DCF, the *Rhodococcus* culture revealed predominantly cells forming *R-*type hydrophobic colonies on the agar medium. The colonies were rough, dull, dry, irregularly-edged and odd-shaped, had a pigmented protruding central area and a non-pigmented flat peripheral part. As we have previously shown, cells that form *R*-type colonies differ from typical *S*-variants by a thickened rigid cell wall with the double excess of the lipid content^58^. The *R-*types are known to exhibit the maximum enzyme concentration in the electron transport chain (cytochromes, flavins), the maximum respiration intensity among dissociants and, ultimately, the maximum growth rate^59^.

### DCF effects on complex physicochemical characteristics of rhodococci (zeta potential, hydrophobicity)

The interaction between DCF and rhodococci resulted in their changed physicochemical characteristics, including the magnitude of ϛ-potential, which reflects quantitatively the electro-surface properties of a cell. Cultivation of Rhodococcus cells during the first 10 days at high (50 mg/L) DCF concentration led to a shift in ϛ-potential from −35.3 ± 2.33 (control) to −31.3 ± 0.83 mV (p < 0.05), indicating the cationic nature of the ecotoxicant (possibly due to the sodium cation present in the DCF molecule and its interaction with the mycolic acid carboxyl groups of the rhodococcal cell wall) and the electrostatic nature of the initial DCF interaction with bacterial cells. On exposure days 20 and 30, a successive shift of ϛ-potential toward the negative values −37.5 ± 2.33 and −47.8 ± 2.57, respectively, was registered. It should be noted that the longer was the cell exposure to DCF, the more negatively charged they became. During this period, a significant number of involutional and lysing cell forms were observed.

Cells grown at low (50 μg/L) DCF concentrations did not statistically differ in ϛ-potential from those cultivated at high DCF concentrations: the numerical values were in similar ϛ-potential range. Negative values of the bacterial cell potential are a consequence of the total negatively charged molecules of lipids, lipoglycans, teichoic and lipoteichoic acids in the bacterial cell wall^60^. Unlike other Gram-positive bacteria, the *Rhodococcus* cell wall has an unusual structure and composition. It is dominated by complex specific lipids composed of 2-alkyl 3-hydroxy branched-chain fatty acids accounting for the formation of an external impermeable lipid barrier^61^. According to our data, the amount of rhodococci-synthesized lipids varied depending on the medium composition. The presence of DCF induced a considerable (almost 2-fold) increase in total lipid content in rhodococci (70.8 ± 4.46% of ADW) compared to that of LB broth-grown cells (43.4 ± 1.72% of ADW). The growth in the lipid component of DCF-cultured rhodococci apparently helps maintain the integrity of the cell membrane and enhances its stability. The data obtained indicate that in the course of DCF biodegradation, the structure of the developing *Rhodococcus* population changed smoothly toward the forms more resistant to toxic DCF effects.

**Figure 6:**
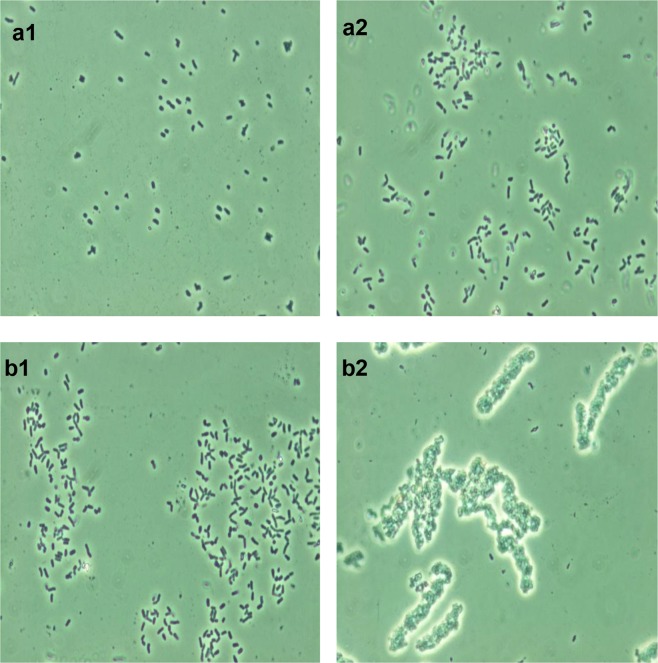
Micrographs of *R. ruber* IEGM 346 grown in LB broth (**a**) for 2 days and in the presence of DCF (50 mg/L) for 10 days (**b**). 1 – without (NH_4_)_2_SO_4_, 2 – with 0.6 M (NH_4_)_2_SO_4_ (x1000).

As the ϛ-zeta potential shift occurred during DCF biodegradation, an intensive formation of cell aggregates was observed. This was consistent with the literature data^62^ that the decrease in the numerical ϛ-potential values of Gram-positive bacteria boosts the cell aggregation phenomenon. The bacterial aggregate formation is not only due to DCF properties and the bacterial surface composition, but also due to the degree of cell surface hydrophobicity. The salt aggregation test showed the hydrophobic interactions between cells to be enhanced in the presence of DCF, which promoted the development of cell aggregates. This is illustrated in Fig. 6: rhodococci produced stable microaggregates when exposed to a relatively low (0.6 M) ammonium sulfate concentration, indicating significant hydrophobicity of their cell surface (Fig. 6b2). It should be noted that at different initial DCF concentrations, a clear tendency (more pronounced at higher DCF concentrations) to cell agglomeration was observed in rhodococci, namely to the growth as discrete, distributed across the liquid mineral medium and visually observed bioconglomerates (globules) of different size and consistency. The obtained results are in agreement with the data of Bouchez-Naïtali *et al*.^63^ reported on high hydrophobicity of flocculating rhodococci grown on hydrophobic substrates and the ability of highly hydrophobic cells to self-aggregate. Such aggregates of hydrophobic cells seem to contribute to cooperative effects of oxidative enzymes on the ecotoxicant.

### Proposed pathways for DCF biodegradation

**Figure 7:**
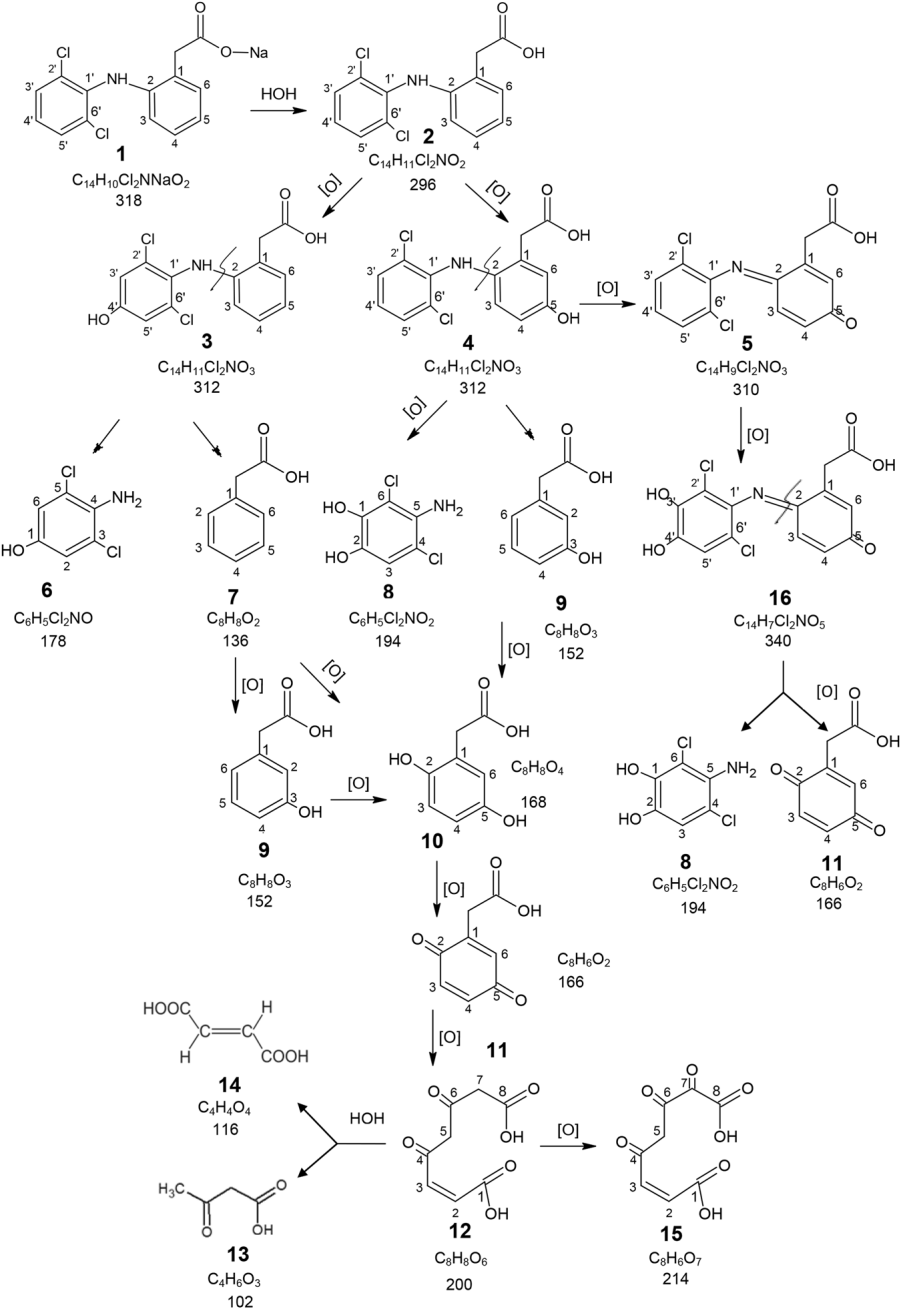
Proposed pathways for DCF biodegradation by *R. ruber* IEGM 346. **1** – 2-[2-(2′,6′-dichloroanilino)phenyl]acetate sodium salt; **2** – 2-[2-(2′,6′-dichloroanilino)phenyl]acetic acid; **3** – 2-[2-(2′,6′-dichloro-4′-hydroxyanilino)phenyl]acetic acid; **4** – 2-[2-(2′,6′-dichloroanilino)-5-hydroxyphenyl]acetic acid; **5** – 2-(1-(5-oxo-cyclohexa-1,3-dienyl-2-(2′,6′-dichloro-phenylimino)acetic acid; **6** – 4-amino-3,5-dichlorophenol; **7** – phenylacetic acid, **8** – 5-amino-4,6-dichlorobenzene-1,2-diol; **9** – 3-hydroxyphenylacetic acid; **10** – 2,5-dihydroxyphenylacetic acid (homogentisic acid); **11** – 2-(*p*-benzoquinone-2)acetic acid; **12** – 4,6-dioxo-oct-2-*trans*-enedioic acid (fumarylacetoacetic acid); **13** – 3-оxobutanoic acid (acetoacetic acid); **14** – *trans*-butenedioic acid (fumaric acid); **15** – 4,6,7-trioxoоct-2-enedioic acid; **16** – 2-[1-(5-oxocyclohexa-1,3-dienyl-2-(3′,4′-dihydroxy-2′,6′-dichlorophenyl)imino]acetic acid.

According to TLC and GC-MS results, bacterial degradation of DCF was accompanied by formation of metabolites. Figure 7 presents a possible scheme for 2-[2-(2′,6′-dichloroanilino)phenyl]acetate sodium salt (**1**) decomposition by *R*. *ruber* IEGM 346. During the first 5–10 days of incubation of rhodococci with high (50 mg/L) DCF concentrations, the primary DCF monohydroxy metabolites 2-[2-(2′,6′-dichloro-4′-hydroxyanilino)phenyl]acetic acid (4′-OH-DCF, **3**), 2-[2-2′,6′-dichloroanilino)-5-hydroxyphenyl]acetic acid (5-OH-DCF, **4**), and also **5** of the benzoquinonimine-type and its dihydroxy derivative (**16**) were detected among DCF (**2**) biodegradation products (Supplementary Figs S1–S3). Afterwards, 2,6-dichloroaniline mono- and dihydroxy derivatives (**6** and **8**) were found in the incubation medium resulting from the C-N bond cleavage at the second carbon atom of the non-chlorinated aromatic ring of compounds **3** and **4**. Additionally, phenylacetic acid (**7**) and its hydroxylated derivative 3-hydroxyphenylacetic acid (**9**) were detected (Supplementary Fig. S4).

At lower (50 μg/L) DCF concentration, the above products were detected in the first 2 days of *Rhodococcus* incubation. On day 4, metabolites spectroscopically similar to a homogentisinic (2,5-dihydroxyphenylacetic) acid, *m/z* 168 (**10**) and its oxidation product 2-(*p*-benzoquinone-2)acetic acid, *m/z* 166 (**11**), and also similar to a fumarylacetoacetic acid, *m/z* 200 (**12**) and its hydrolysis products acetoacetic acid, *m/z* 102, and fumaric acid, *m/z* 116 (**13** and **14**, respectively), were recorded in the medium (Supplementary Figs S5–S7). The presence of phenylacetic acid (Rf 0.64) in the intermediate stage of DCF biodegradation, and fumaric acid (Rf 0.83) at the end of DCF biodegradation was confirmed by TLC results (Supplementary Table S1).

Additionally, an intense peak of **15** (*m/z* 214) was observed; this compound apparently resulted from the oxidation of **12** at the seventh carbon atom due to the mobile hydrogen atoms between the carboxyl and ketone groups (Supplementary Fig. S7). The presence of the ketone group and the double bond was confirmed using semicarbazide hydrochloride and bromine water. In the IR spectra of dry residues of DCF metabolites, the characteristic absorption bands of phenol hydroxyl at 3,400 cm^−1^ at the beginning of the experiment and ketone carbonyl at 1,670–1,680 cm^−1^ at the end of the experiment were observed. At the end of fermentation (on experimental day 6), DCF and the above compounds were not detected in the incubation medium of rhodococci, indicating the further metabolite transformation. The controls showed no DCF metabolites.

## Discussion

Screening of 104 *Rhodococcus* strains from the IEGM collection resulted in selection of *R*. *ruber* IEGM 346 highly tolerant (MIC 200 mg/L) to DCF. Within 6 days, this strain completed the ecotoxic DCF (50 μg/L) degradation with glucose (0.5%) added to ensure significant cell growth, and rhodococci pre-incubation at low (5 μg/L) DCF concentration to stimulate the multipurpose oxygenase complex.

DCF exposure on rhodococci was shown to lead to morphological anomalies of cells manifested in the changed cell shape and size, and surface roughness. In the presence of DCF, there was a significant cell aggregation, as well as a change in ζ-potential, an increase in hydrophobicity and total cellular lipid content.

Knowledge on the ecopollutant metabolism is an important factor in assessing its safety. With glucose added, rhodococci decompose DCF via three proposed pathways for primary hydroxy metabolites (Fig. 7). Pathway I: 4′-ОН-DCF (**3**) decomposes due to C-N bond cleavage at the second carbon atom in the non-chlorinated aromatic ring to form 4-amino-3,5-dichlorophenol (**6**) and phenylacetic acid (**7**). The latter is hydroxylated at positions 2 and 5 of the aromatic ring to homogentisinic (2,5-dihydroxyphenylacetic) acid (**10**), which is oxidized to 2-(*p*-benzoquinone-2)acetic acid (**11**). Subsequent oxidation of **11** leads to the quinone ring cleavage at positions 5 and 6 to form fumarylacetoacetic acid (**12**), which is hydrolyzed to form acetoacetic acid (**13**) and fumaric acid (**14**). Pathway II: 5-ОН-DCF (**4**) is decomposed owing to C-N bond cleavage at the second carbon atom in the non-chlorinated aromatic ring to form 5-amino-4,6-dichlorobenzene-1,2-diol (**8**) and 3-hydroxyphenylacetic acid (**9**). Compound **9** is oxidized to form homogentisinic acid (**10**), which is further converted to 2-(*p*-benzoquinone-2)acetic acid (**11**). Subsequent transformations of **11** via the pathway I lead to acetoacetic acid (**13**) and fumaric acid (**14**). Pathway III: 5-OH-DCF (**4**) is oxidized to form benzoquinonimine (**5**), which is hydroxylated to form a dihydroxy compound (**16**). The azomethine bond in **16** is hydrolyzed to form **8** and quinone (**11**), with concurrent structure oxidation at the second carbon atom. Compound **11** undergoes the above changes via pathway I.

Particularly noteworthy are two events: (i) the C–N bond cleavage in DCF structure to form phenylacetic acid, and (ii) the possible quinone ring opening to form fumarylacetoacetic acid and its hydrolysis products acetoacetic and fumaric acids, which can be considered as DCF detoxification products. Further research is needed to confirm these findings.

In this study, 16 metabolites produced by *R*. *ruber* IEGM 346 were identified, four of which (4′-OH-DCF, 5-OH-DCF and two benzoquinone imine-type compounds) are similar to those obtained in earlier studies of DCF metabolism by the alphaproteobacterium *Labrys portucalensis* F11^45^, and two metabolites (4′-OH-DCF and 5-OH-DCF) obtained using Gram-positive bacteria *Actinoplanes*^40^. However, all the above metabolites retained the C-N bond at the second carbon atom in the non-chlorinated aromatic ring of DCF. At the same time, there was no evidence confirming the aromatic ring opening in the structures of compounds formed.

The obtained fundamental data elucidate the ecological role of rhodococci in detoxification of pharma pollutants and underpin the implementation of innovative technical solutions for advanced pharmaceutical sewage treatment. Because *R*. *ruber* IEGM 346 converts DCF to form lots of metabolites that are relatively easy to detect, isolate and identify, and also shows similarity to DCF metabolism in mammals, this strain can be used as a bacterial model, alternative to mammalian model systems^64^, to study the metabolism of polycyclic aromatic pharmaceutical compounds and their metabolites’ synthesis. Additionally, rhodococci have a high level of expression of P450s (CYPs), enzymes^2^ involved in drug metabolism by changing the functional groups of the parent molecule.

## Supplementary information

Supplementary Information

## Data Availability

The data generated during and/or analyzed during the current study are available from the corresponding author on reasonable request.

## References

[1] de Carvalho, C. C. C. R. Adaptation of *Rhodococcus* to organic solvents. In *Biology of* Rhodococcus (ed. Alvarez, H. M.) 109–131 (Springer-Verlag, 2010).

[2] Ivshina, I. B., Kuyukina, M. S. & Krivoruchko, A. V. Hydrocarbon-oxidizing bacteria and their potential in eco-biotechnology and bioremediation. In *Microbial Resources* (ed. Kurtböke, I.) 121–148 (Elsevier Inc., 2017).

[3] Kuyukina, M. S. & Ivshina, I. B. Application of *Rhodococcus* in bioremediation of contaminated environments. In *Biology of**Rhodococcus* (ed. Alvarez, H. M.) 231–262 (Springer-Verlag, 2010).

[4] Larkin MJ, Kulakov LA, Allen CCR (2006). Biodegradation by members of the genus *Rhodococcus*: biochemistry, physiology, and genetic adaptation. Adv. Appl. Microbiol..

[5] Gauthier H, Yargeau V, Cooper DG (2010). Biodegradation of pharmaceuticals by *Rhodococcus rhodochrous* and *Aspergillus niger* by co-metabolism. Sci. Total Environ..

[6] Evangelista S, Yargeau V, Cooper DG (2008). The recalcitrance of clofibric acid to microbial degradation. WIT Trans. Ecol. Environ..

[7] Kim YU (2007). Steroid 9α-hydroxylation during testosterone degradation by resting *Rhodococcus equi* cells. Arch. Pharm. (Weinheim)..

[8] Larcher S, Yargeau V (2013). Biodegradation of 17α-ethinylestradiol by heterotrophic bacteria. Environ. Pollut..

[9] O’Grady D, Evangelista S, Yargeau V (2009). Removal of aqueous 17α-ethinylestradiol by *Rhodococcus* species. Environ. Eng. Sci..

[10] Yoshimoto T (2004). Degradation of estrogens by *Rhodococcus zopfii* and *Rhodococcus equi* isolates. Appl. Environ. Microbiol..

[11] Ivshina IB, Rychkova MI, Vikhareva EV, Chekryshkina LA, Mishenina II (2006). Catalysis of the biodegradation of unusable medicines by alkanotrophic rhodococci. Appl. Biochem. Microbiol..

[12] Ivshina IB, Vikhareva EV, Richkova MI, Mukhutdinova AN, Karpenko JN (2012). Biodegradation of drotaverine hydrochloride by free and immobilized cells of *Rhodococcus rhodochrous* IEGM 608. World J. Microbiol. Biotechnol..

[13] Ivshina IB (2014). Drotaverine hydrochloride degradation using cyst-like dormant cells of *Rhodococcus ruber*. Curr. Microbiol..

[14] aus der Beek T (2016). Pharmaceuticals in the environment – global occurrences and perspectives. Environ. Toxicol. Chem..

[15] Fatta-Kassinos D, Meric S, Nikolaou A (2011). Pharmaceutical residues in environmental waters and wastewater: current state of knowledge and future research. Anal. Bioanal. Chem..

[16] Zhang Y, Geißen SU, Gal C (2008). Carbamazepine and diclofenac: removal in wastewater treatment plants and occurrence in water bodies. Chemosphere.

[17] Sui Q (2015). Occurrence, sources and fate of pharmaceuticals and personal care products in the groundwater: a review. Emerg. Contam..

[18] Yang L (2017). Occurrence, distribution, and attenuation of pharmaceuticals and personal care products in the riverside groundwater of the Beiyun River of Beijing, China. Environ. Sci. Pollut. Res..

[19] Alygizakis NA (2016). Occurrence and spatial distribution of 158 pharmaceuticals, drugs of abuse and related metabolites in offshore seawater. Sci. Total Environ..

[20] Huebner M, Weber E, Niessner R, Boujday S, Knopp D (2015). Rapid analysis of diclofenac in freshwater and wastewater by a monoclonal antibody-based highly sensitive ELISA. Anal. Bioanal. Chem..

[21] Nebot C, Falcon R, Boyd KG, Gibb SW (2015). Introduction of human pharmaceuticals from wastewater treatment plants into the aquatic environment: a rural perspective. Environ. Sci. Pollut. Res..

[22] UNESCO and HELCOM. *Pharmaceuticals in the aquatic environment of the Baltic Sea region.* (UNESCO Publishing, 2017).

[23] Dasenaki ME, Thomaidis NS (2015). Multianalyte method for the determination of pharmaceuticals in wastewater samples using solid-phase extraction and liquid chromatography-tandem mass spectrometry. Anal. Bioanal. Chem..

[24] Kot-Wasik A, Jakimska A, Śliwka-Kaszyńska M (2016). Occurrence and seasonal variations of 25 pharmaceutical residues in wastewater and drinking water treatment plants. Environ. Monit. Assess..

[25] Rivera-Jaimes JA (2018). Study of pharmaceuticals in surface and wastewater from Cuernavaca, Morelos, Mexico: occurrence and environmental risk assessment. Sci. Total Environ..

[26] Singh KP, Rai P, Singh AK, Verma P, Gupta S (2014). Occurrence of pharmaceuticals in urban wastewater of north Indian cities and risk assessment. Environ. Monit. Assess..

[27] Vieno N, Sillanpää M (2014). Fate of diclofenac in municipal wastewater treatment plant – a review. Environ. Int..

[28] Khan U, Nicell J (2015). Human health relevance of pharmaceutically active compounds in drinking water. AAPS J..

[29] Simazaki D (2015). Occurrence of selected pharmaceuticals at drinking water purification plants in Japan and implications for human health. Water Res..

[30] Bouju H (2016). Elucidation of biotransformation of diclofenac and 4′hydroxydiclofenac during biological wastewater treatment. J. Hazard. Mater..

[31] Gröning J (2007). Transformation of diclofenac by the indigenous microflora of river sediments and identification of a major intermediate. Chemosphere.

[32] Barra Caracciolo A, Topp E, Grenni P (2015). Pharmaceuticals in the environment: biodegradati on and effects on natural microbial communities. A review. J. Pharm. Biomed. Anal..

[33] González-Alonso S (2017). Occurrence of pharmaceutical, recreational and psychotropic drug residues in surface water on the northern Antarctic Peninsula region. Environ. Pollut..

[34] Acuña V (2015). Balancing the health benefits and environmental risks of pharmaceuticals: diclofenac as an example. Environ. Int..

[35] D. 495/2015/EU. (2015). Commission Implementig Decision (EU) 2015/495 of 20 March 2015 establishing a watch list of substances for Union-wide monitoring in the field of water policy pursuant to Directive 2008/105/EC of the European Parliament and of the Council. Off. J. Eur. Union..

[36] Fischer K, Kühnert M, Gläser R, Schulze A (2015). Photocatalytic degradation and toxicity evaluation of diclofenac by nanotubular titanium dioxide-PES membrane in a static and continuous setup. RSC Adv..

[37] Márquez Brazón E, Piccirillo C, Moreira IS, Castro PML (2016). Photodegradation of pharmaceutical persistent pollutants using hydroxyapatite-based materials. J. Environ. Manage..

[38] Schröder P (2016). Status of hormones and painkillers in wastewater effluents across several European states – considerations for the EU watch list concerning estradiols and diclofenac. Environ. Sci. Pollut. Res..

[39] Domaradzka D, Guzik U, Wojcieszyńska D (2015). Biodegradation and biotransformation of polycyclic non-steroidal anti-inflammatory drugs. Rev. Environ. Sci. Biotechnol..

[40] Osorio-Lozada A, Surapaneni S, Skiles GL, Subramanian R (2008). Biosynthesis of drug metabolites using microbes in hollow fiber cartridge reactors: case study of diclofenac metabolism by *Actinoplanes* species. Drug Metab. Dispos..

[41] Bessa VS, Moreira IS, Tiritan ME, Castro PML (2017). Enrichment of bacterial strains for the biodegradation of diclofenac and carbamazepine from activated sludge. Int. Biodeterior. Biodegrad..

[42] Kosjek T, Heath E, Pérez S, Petrović M, Barceló D (2009). Metabolism studies of diclofenac and clofibric acid in activated sludge bioreactors using liquid chromatography with quadrupole-time-of-flight mass spectrometry. J. Hydrol..

[43] Langenhoff A (2013). Microbial removal of the pharmaceutical compounds ibuprofen and diclofenac from wastewater. Biomed Res. Int..

[44] Tiehm A (2011). Biodegradation of pharmaceutical compounds and their occurrence in the Jordan valley. Water Resour. Manag..

[45] Moreira IS (2018). Biodegradation of diclofenac by the bacterial strain *Labrys portucalensis* F11. Ecotoxicol. Environ. Saf..

[46] Aissaoui S, Ouled-Haddar H, Sifour M, Harrouche K, Sghaier H (2017). Metabolic and co-metabolic transformation of diclofenac by *Enterobacter hormaechei* D15 isolated from activated sludge. Curr. Microbiol..

[47] Stylianou K, Hapeshi E, Vasquez MI, Fatta-Kassinos D, Vyrides I (2018). Diclofenac biodegradation by newly isolated *Klebsiella* sp. KSC: microbial intermediates and ecotoxicological assessment. J. Environ. Chem. Eng..

[48] Catalogue of Strains of Regional Specialised Collection of Alkanotrophic Microorganisms. Available at, http://www.iegmcol.ru/strains/index.html.

[49] Clinical and Laboratory Standards Institute. Performance standards for antimicrobial susceptibility testing. CLSI supplement M100. 27th ed. CLSI. (Wayne, PA, 2017).

[50] Lindahl M, Faris A, Wadström T, Hjertén S (1981). A new test based on ‘salting out’ to measure relative hydrophobicity of bacterial cells. BBA – Gen. Subj..

[51] Mattos-Guaraldi AL, Formiga LCD, Andrade AFB (1999). Cell surface hydrophobicity of sucrose fermenting and nonfermenting *Corynebacterium diphtheriae* strains evaluated by different methods. Curr. Microbiol..

[52] Dastidar SG, Ganguly K, Chaudhuri K, Chakrabarty AN (2000). The anti-bacterial action of diclofenac shown by inhibition of DNA synthesis. Int. J. Antimicrob. Agents.

[53] Salem-Milani A (2013). Antibacterial effect of diclofenac sodium on *Enterococcus faecalis*. J. Dent. (Tehran).

[54] Dutta NK (2004). Antimycobacterial activity of the antiinflammatory agent diclofenac sodium, and its synergism with streptomycin. Brazilian J. Microbiol..

[55] Neumann G (2005). Cells of *Pseudomonas putida* and *Enterobacter* sp. adapt to toxic organic compounds by increasing their size. Extremophiles.

[56] Korshunova IO, Pistsova ON, Kuyukina MS, Ivshina IB (2016). The effect of organic solvents on the viability and morphofunctional properties of *Rhodococcus*. Appl. Biochem. Microbiol..

[57] Cheremnykh KM, Luchnikova NA, Grishko VV, Ivshina IB (2018). Bioconversion of ecotoxic dehydroabietic acid using *Rhodococcus* actinobacteria. J. Hazard. Mater..

[58] Kuyukina MS, Ivshina IB, Rychkova MI, Chumakov OB (2000). Effect of cell lipid composition on the formation of nonspecific antibiotic resistance in alkanotrophic rhodococci. Microbiology.

[59] Iwabuchi N, Sunairi M, Anzai H, Nakajima M, Harayama S (2000). Relationships between colony morphotypes and oil tolerance in *Rhodococcus rhodochrous*. Appl. Environ. Microbiol..

[60] Wilson WW, Wade MM, Holman SC, Champlin FR (2001). Status of methods for assessing bacterial cell surface charge. J. Microbiol. Methods.

[61] Gibson KJC (2003). Structural and functional features of *Rhodococcus ruber* lipoarabinomannan. Microbiology.

[62] Kłodzińska E (2010). Effect of zeta potential value on bacterial behavior during electrophoretic separation. Electrophoresis.

[63] Bouchez-Naïtali M, Blanchet D, Bardin V, Vandecasteele JP (2001). Evidence for interfacial uptake in hexadecane degradation by *Rhodococcus equi*: the importance of cell flocculation. Microbiology.

[64] Asha S, Vidyavathi M (2009). *Cunninghamella* – a microbial model for drug metabolism studies – a review. Biotechnol. Adv..

