# Supplementary material for: Features of diclofenac biodegradation by Rhodococcus ruber IEGM 346
Source: Sci Rep. 2019 Jun 24;9:9159. doi: 10.1038/s41598-019-45732-9 (PMC6591480; doi:10.1038/s41598-019-45732-9)
Supplement: Supplementary file 1 — Supplementary Information [file 41598_2019_45732_MOESM1_ESM.pdf]

Features of diclofenac biodegradation by *Rhodococcus ruber* IEGM 346

Irina B. Ivshina [a,b*], **Elena A. Tyumina** [b], **Maria V. Kuzmina**[c], **Elena V. Vikhareva** [c]

[a] *Institute of Ecology and Genetics of Microorganisms, Ural Branch of the Russian Academy of Sciences, 13 Golev Street, 614081 Perm, Russia*

[b] *Perm State National Research University, 15 Bukirev Street, 614990 Perm, Russia*

[c] *Perm State Pharmaceutical Academy, 2 Polevaya Street, 614990 Perm, Russia*

***** Corresponding author at: Institute of Ecology and Genetics of Microorganisms, Ural Branch of the Russian Academy of Sciences, 13 Golev Street, 614081 Perm, Russia. Tel.: +7(342)2808114. Fax: +7(342)2809211

E-mail address: ivshina@iegm.ru (I. B. Ivshina)

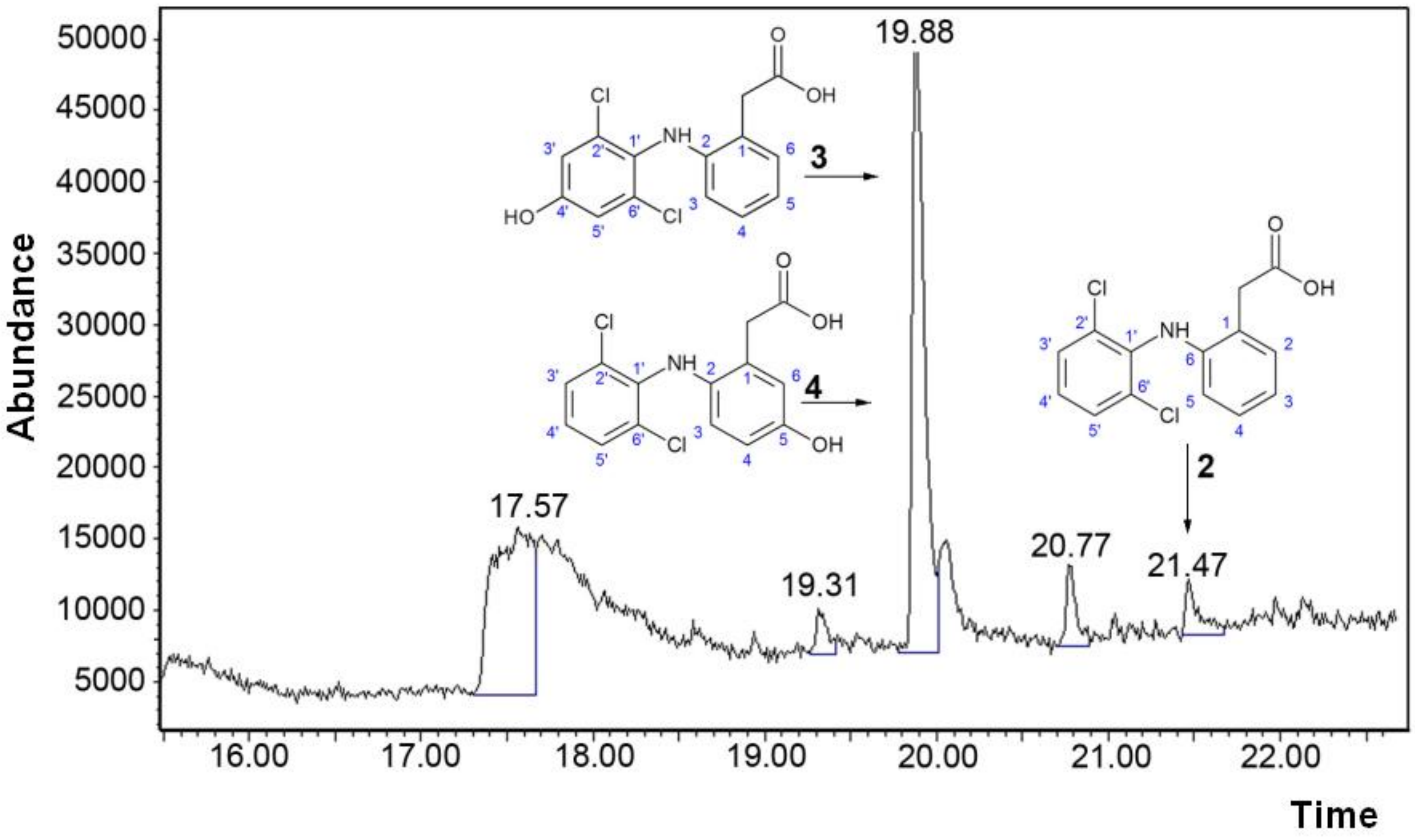

Supplementary Figure S1. Chromatogram of DCF metabolites detected in the post-fermentation medium of rhodococci at the beginning of the biodegradation process. **2** – 2-[2-(2',6'-dichloroanilino)phenyl]acetic acid; **3** – 2-[2-(2',6'-dichloro-4'-hydroxyanilino)phenyl]acetic acid; **4** – 2-[2-(2',6'-dichloroanilino)-5-hydroxyphenyl]acetic acid.

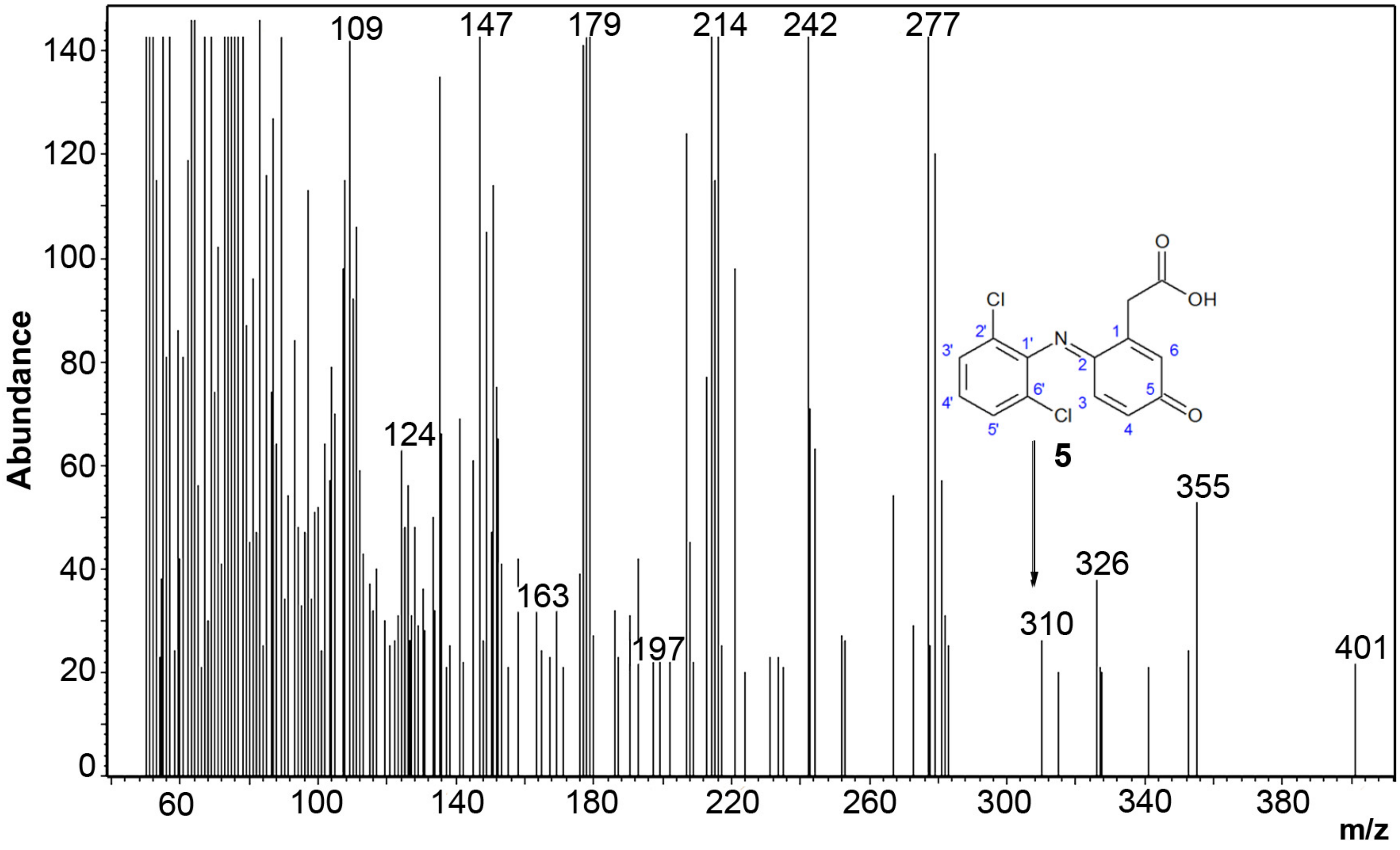

Supplementary Figure S2. Mass-spectrum of (**5**) 2-(1-(5-oxo-cyclohexa-1,3-dienyl-2-(2',6'-dichloro-phenylimino)acetic acid, *m/z*=310.0.

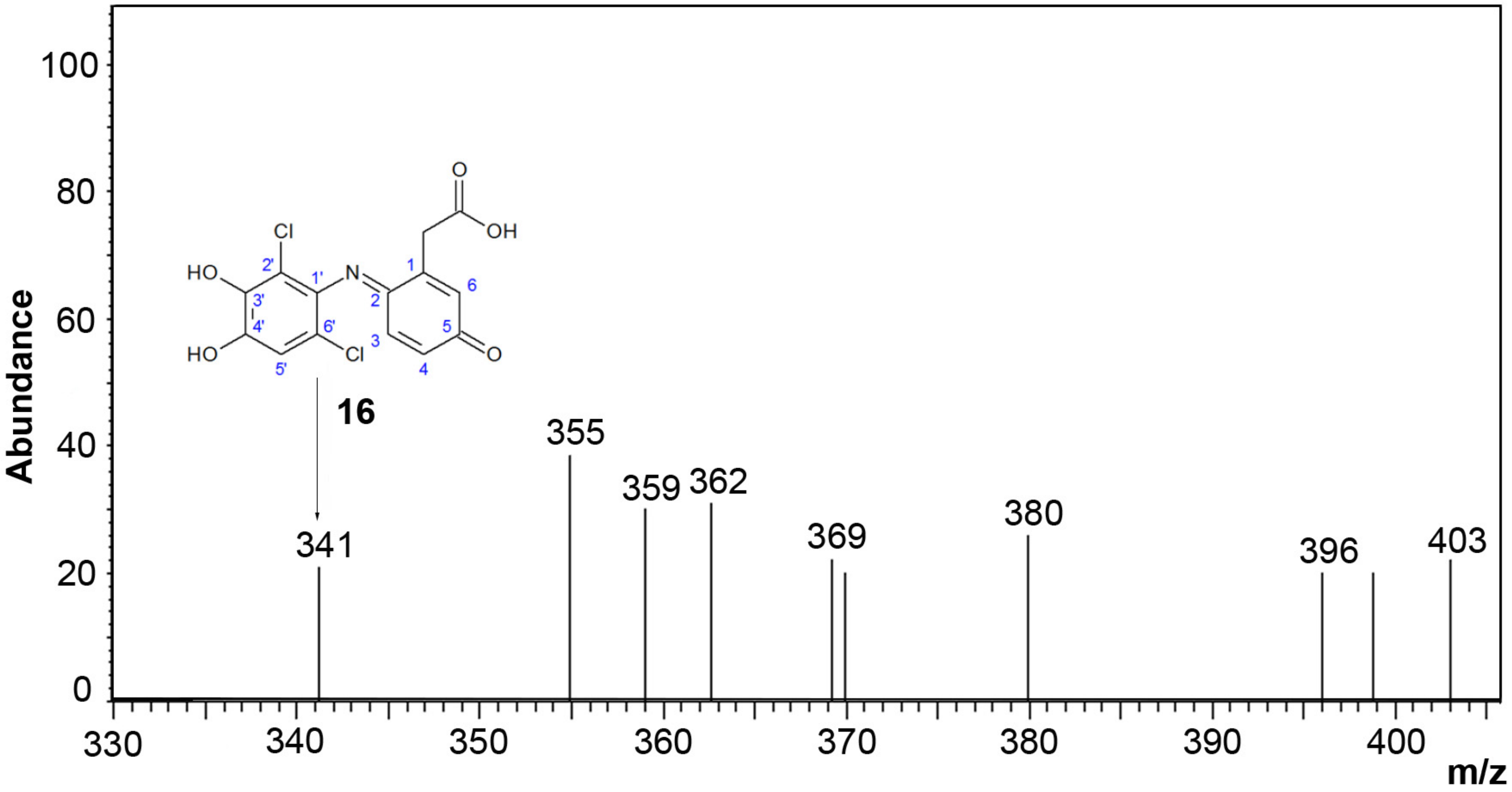

Supplementary Figure S3. Mass-spectrum of (**16**) 2-[1-(5-oxocyclohexa-1,3-dienyl-2-(3',4'-dihydroxy-2',6'-dichlorophenyl)imino]acetic acid, *m/z*=340.0.

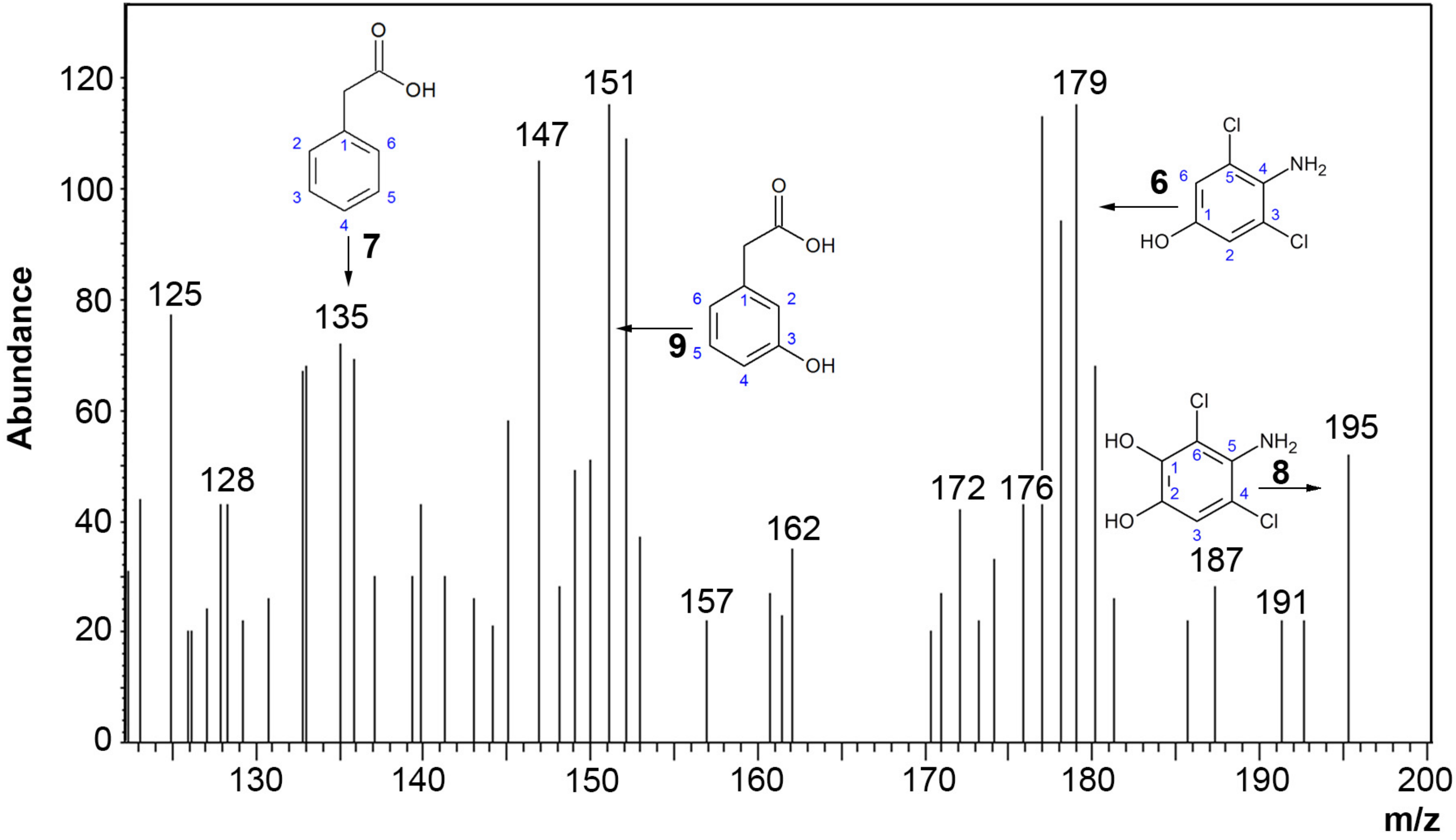

Supplementary Figure S4. Mass-spectra of (**6**) 4-amino-3,5-dichlorophenol, *m/z*=178.0; (**7**) phenylacetic acid, *m/z*=136; (**8**) 5-amino-4,6-dichlorobenzene-1,2-diol, *m/z*=194.0; (**9**) 3-hydroxyphenylacetic acid, *m/z*=152.0.

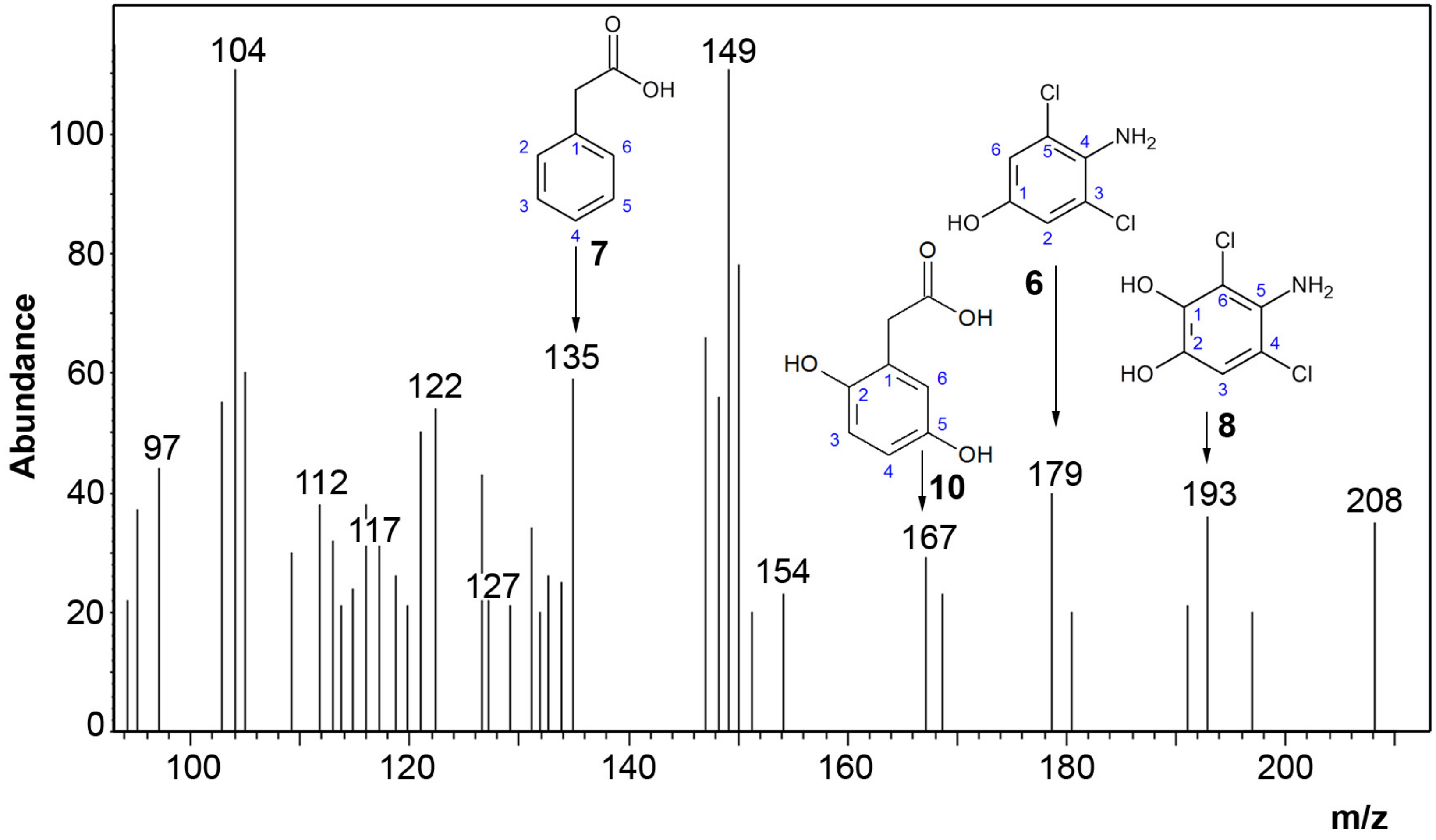

Supplementary Figure S5. Mass-spectra of (**6**) 4-amino-3,5-dichlorophenol, *m/z*=178.0; (**7**) phenylacetic acid, *m/z*=136; (**8**) 5-amino-4,6-dichlorobenzene-1,2-diol, *m/z*=194.0; (**9**) 3-hydroxyphenylacetic acid, *m/z*=152.0; (**10**) 2,5-dihydroxyphenylacetic acid (homogentisic acid), *m/z*=167.0.

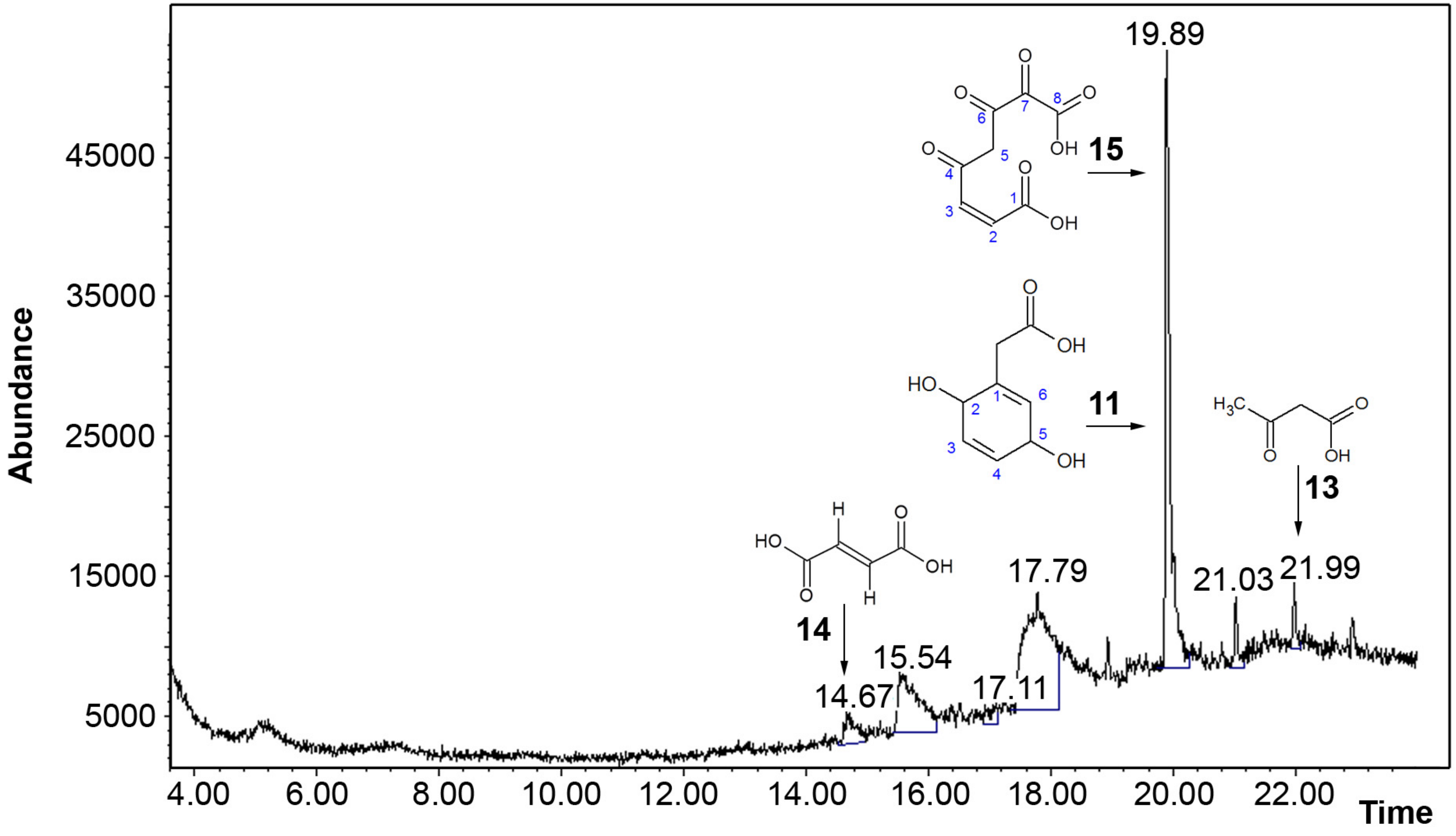

Supplementary Figure S6. Chromatogram of DCF metabolites detected in the post-fermentation medium of rhodococci at the end of the biodegradation process. **11** – 2-(*p*-benzoquinone-2)acetic acid, **13** – 3-oxobutanoic acid (acetoacetic acid), **14** – *trans*-butenedioic acid (fumaric acid), **15** – 4,6,7-trioxooct-2-enedioic acid.

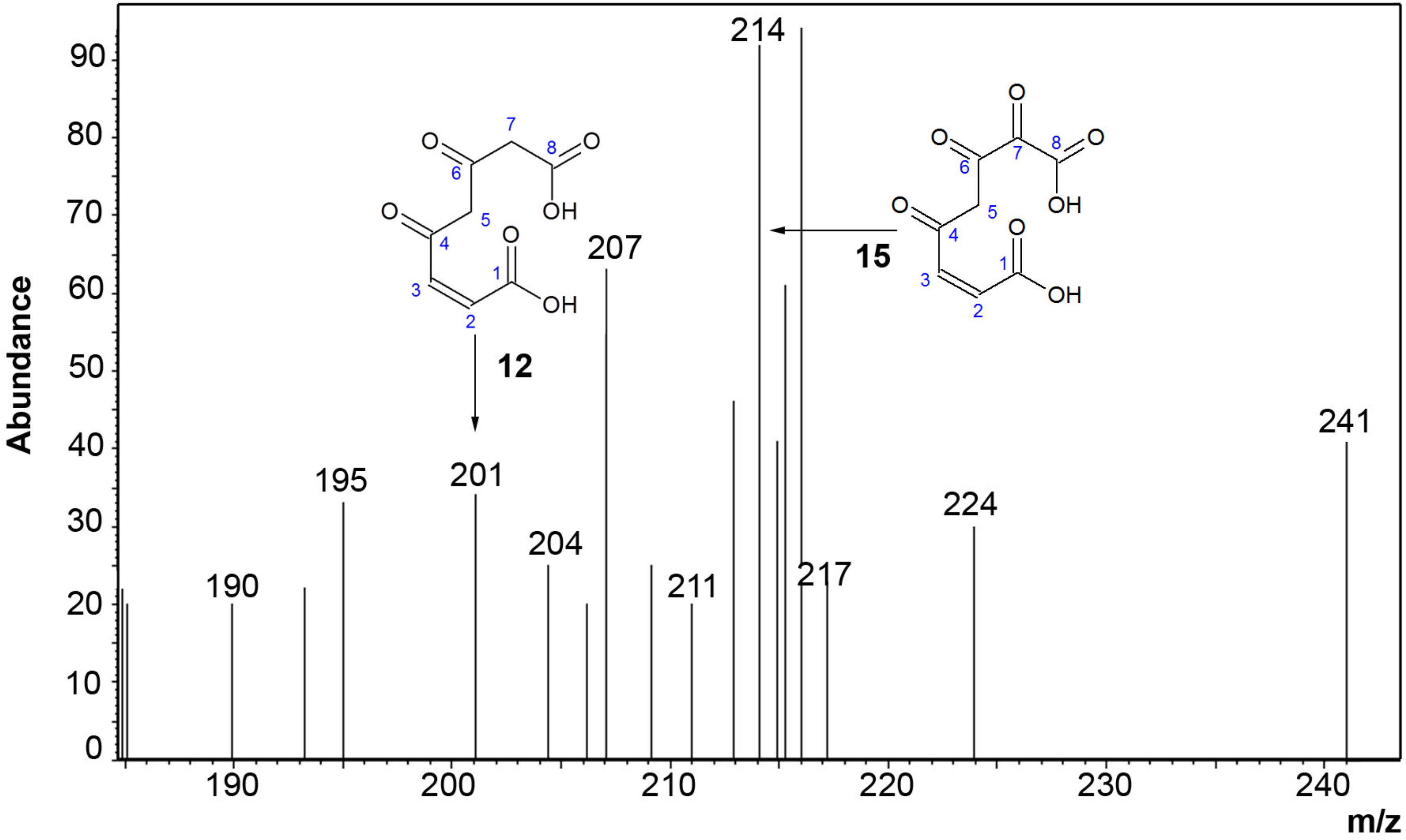

Supplementary Figure S7. Mass-spectra of (**12**) 4,6-dioxo-oct-2-*trans*-enedioic acid (fumarylacetoacetic acid), *m/z*=200.0; (**15**) 4,6,7-trioxooct-2-enedioic acid, *m/z*=214.0.

Supplementary Table S1. Rf values of DCF and its biodegradation products in culture liquids of *R. ruber* IEGM 346.

No.	Test substance	Chemical structure	R_f value
1	Fumaric acid	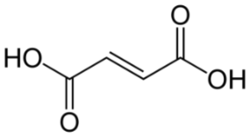<br>**Trans isomer**	**0.83**
2	Phenylacetic acid	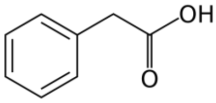	**0.64**
3	DCF	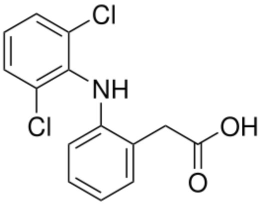	**0.65**
4	Culture fluid containing 50% of residual DCF		**0.83**<br>**0.65**<br>**0.64**
5	The culture fluid containing only DCF metabolites (0% diclofenac)		**0.83**

Note. Each value of Rf is the average of three variables.

Identification of DCF biodegradation products using TLC in the system of benzene: 95% ethanol: glacial acetic acid (45:8:4, v/v/v) showed that fumaric acid was present in sample No. 5 (culture fluid containing only DCF metabolites).
